# Determinants of preterm birth among mothers delivered in Central Zone Hospitals, Tigray, Northern Ethiopia

**DOI:** 10.1186/s13104-019-4307-z

**Published:** 2019-05-14

**Authors:** Tesfay Berhe, Hailay Gebreyesus, Haftom Desta

**Affiliations:** 1 Department of Public Health, College of Health Sciences, Aksum University, P. O. Box: 298, Aksum, Ethiopia; 2 Health Education and Promotion, Department of Public Health, College of Health Science, Aksum University, Aksum, Ethiopia; 3 Department of Psychiatric Nursing, College of Health Sciences, Aksum University, Aksum, Ethiopia

**Keywords:** Determinants, Preterm birth, Pregnant women, Gestational age, Newborn

## Abstract

**Objective:**

Preterm deliveries were responsible for 3.1 million neonatal deaths that occurred globally in 2010, of them 35% were directly related to preterm. This study was aimed to assess the determinants of preterm birth among women delivered of Central zone. A facility-based cross-sectional study was carried out from March to May 2018, in four randomly selected hospitals of Central zone. A total of 413 participants were interviewed using a pre-tested structured questionnaire. Data were entered into Epi-info Version 3.5.3 and then exported to SPSS Version 21 for analysis. Bivariate and multivariate logistic regression analysis was carried out to assess the association. Statistical significance was declared by 95% confidence interval of the odds ratio.

**Result:**

From the total (413) participants, 78.7% of participants were in the age group of 20-34 years and in this study the prevalence of preterm birth was 12.8%. Factors like, marital status of unmarried (AOR = 5.21, 95% CI 1.8–15.075), mid upper arm circumference (MUAC) (< 11 cm) (AOR = 2.42, 95% CI 1.204–4.851) were independent predictors of preterm birth among women delivered in the study area.

## Introduction

Preterm birth defined as live births before 37 completed weeks of gestational age [1–3]. From the 130 million babies born each year globally, approximately 15 million are born preterm and 60% of preterm is occur in developing countries [4–6]. The risk of neonatal death in African baby is higher than European baby by at least 12 times due to complications of preterm birth. By comparing of economic status, over 90% of extremely preterm babies (< 28 weeks) born in low-income countries die within the first few days of life while only less than 10% of babies of this gestation die in high-income settings [5, 7].

In Ethiopia 320,000 preterm births are happening every year [8]. Under-5 mortality rates are reducing in many countries and Ethiopia also achieved the millennium development goal on child mortality reduction. However, neonatal mortality rate have been shown much less progress. Neonatal deaths account for an increasing proportion of child deaths worldwide (41% in 2015) much higher than the 38% in 2000 [9–12]. About 15 million babies born too early each year, that is more than one in ten births. Every year around one million children die due to the consequence of prematurity birth [3, 8, 13].

Even though, many women have a premature birth, its risk factor is idiopathic. But it can be occurred due to many risk factors. These could be: history of premature birth, multiple pregnancies, short spacing between pregnancies (mainly less than 6 months), troubles with the uterus, substance use like drug or cigarette, low intake of nutrients, in adequate increasing of weight during the period of pregnancy, complication due to infections and some of the non-communicable diseases [14].

From the total, about 85% of preterm babies are delivered between 32 week and 37 weeks of gestation. These babies do not need intensive care for their survival. In Ethiopia, which is one of the developing country, about 320,000 babies are born very early each year and around 24,400 less than 5 year children die due to direct effect of preterm [8, 15]. The main cause of neonatal morbidity and mortality and the occurrence of preterm birth are considered a complex public health condition. With variable incidence in several countries, it has grown markedly in the last decades [16]. Therefore, it is essential to establish the prevalence and causes of this condition in order to propose prevention actions.

## Main text

### Methods

#### Study setting

The study was carried out at public hospitals of Central zone which is found in Tigray regional state. It is located at 1040 km far from Addis Ababa, capital city of Ethiopia to the North. The total population of the zone is 1,347,212, of which about 686,161 are female (according to zonal 2017 official report). Administratively, it is divided into 8 districts and has nine hospitals.

#### Study design and population

This study was done using a facility-based cross-sectional study design from March to May 2018. The respondents of this study were women who gave births a baby in the hospitals and who lived in the central zone for at least 6 months were included in the study. Those women who did not responses appropriately the interview due to physical or mental illness were excluded from the study.

#### Sample size and sampling technique

The sample size of this study was calculated using a single proportion formula as follow:

$${\text{n}} = \left( {{\text{z}}\upalpha^{2} } \right){\text{p}}\left( {1 - {\text{p}}} \right)/{\text{d}}^{2}$$

where n = number of the study subjects (sample size), Z = standardized normal distribution value for the 95% confidence level (1.96), d = Margin of error taken (0.05), p = prevalence of preterm birth from previous study (14.3% [15]. Based on the above assumptions, by using of 2 design effect and 10% non-response, the final sample size becomes 413.

In this study four hospitals were randomly selected from the nine hospitals of Central zone. Later, systematic sampling technique was being employed for selection of the participants. Study subjects were selecting every two mothers until the sampled population fills. Selection of the first sample was taken through simple random lottery method. All mothers who gave births in the selected hospitals during the specific study period were used as the study population.

#### Operational definition

Districts: small Administrative sub-zones.

History of poor obstetric outcome: mothers who had history of LBW, preterm birth, still birth, perinatal death and abortion.

Kebelles: small administrative unit in Ethiopia.

Preterm birth: live births before 37 completed weeks of gestational age.

#### Data collection tools and procedure

A structured, pretested and quantitative interviewer administered questionnaire was adopted by reviewing different literatures. The questionnaire was grouped into six categories; socio-demographic information, gynecological and obstetric, new born, medical disorder, nutritional and heath care related factors. The questionnaire was prepared in English and then translated into Tigrigna version and then translated back to English for its consistency and completeness.

To maintain data quality, data were collected by Clinical midwife and MPH in public health supervisors. Both of data collectors and supervisors were trained for 2 days to ensure the quality of data including clarification of questions to make simple and easily understandable, to use recommended ways of sampling technique and to inform the study subjects based on the consent. Four clinical midwives were collected the data by face to face interview immediately after birth and retrieved the remaining data from medical records. Two MPH in public health supervised the data collector’s and they had been communicated daily with the authors. The questionnaire was tested for its clarity, consistency and unambiguous language in the one selected hospital which was not included in the study and appropriate modification had done based on findings. Supervisors and authors were strictly followed the data collection process and filled questionnaire were reviewed daily for completeness and consistency.

#### Data processing and analysis procedures

After data collection was made, each questionnaire had checked manually for its completeness before entering into software. The data was entered, cleaned and coded using Epi-data version 3.5.3 and data analysis was done using SPSS version 21. The data was described using frequency tables and descriptive statistics. The association between dependent and independent factors was analyzed using binary logistic regression analysis with crude Odds Ratio along with 95% confidence interval, then these factors with *p* value < 0.25 analyzed using multivariate logistic regression analysis to determine the associated factors with preterm birth and to control confounding factors. In multivariate analysis, variables having p-value less than 0.05 and adjusted odds ratio with 95% CI were considered as significantly associated with the outcome variables.

### Result

#### Socio-demographic characteristics of respondents

Out of the 413 mothers, a total of 410 (99%) mothers participated in the study. Three hundred six (74.1%) were in the age group between 20 and 34 years. About 390 (94.4%) of the participants were Orthodox Christian followers and 397 (96.1%) were married. Regarding maternal educational status 258 (62.4%) were secondary and above and 198 (48%) were house wife. About 187 (45.3%) of the participants their family monthly income were in the range of 89–178.5$ (Table 1).

**Table 1:** Distribution of socio-demographic characteristics of respondents in Central zone, Northern Ethiopia, 2018

Variables	Number	Percent
Maternal age		
< 20	48	11.6
20–34	325	78.7
35+	40	9.7
Religion		
Orthodox	390	94.4
Muslim	23	5.6
Marital status		
Single	16	3.9
Married	39	96.1
Maternal educational status		
Illiterate	56	13.6
Elementary	99	24
Secondary and above	258	62.4
Maternal occupation		
Housewife	198	48
Daily laborer	26	6.3
Employed	97	23.5
Merchant	92	22.2
Income		
< 89$	101	24.5
89$–178.5$	187	45.3
> 178.5$	125	30.2

#### Gynecological and obstetric and new born characteristics of respondents

From the total respondents 293 (70.9%) were multipara. Most of the mothers 332 (80.4%) received ANC four times and above, 330 (80%) initiated ANC at first trimester of the current pregnancy, 374 (90.6%) were taken iron supplementation, 290 (77.5%) were taken iron supplementation for less 3 months and from the pregnancies 383 (92.7%) were planned. Three hundred fifty-five (86%) were gave birth between 37 and 42 weeks of gestational age, where as 53 (12.8%) and 5 (1.2%) were gave birth in < 37 weeks and > 42 weeks, respectively. Majority of mothers 287 (69.5%) were counseled to take additional diet, 286 (69.3%) were took additional diet during current pregnancy. Four hundred two (97.3%) mothers were not faced any pregnancy related complication during current pregnancy. Of the total births 400 (96.9%) were live births and 13 (3.1%) were stillbirths. Regarding birth weight 59 (14.7%) newborns were low birth weight and related to maturity 51 (12.3%) were preterm. Besides 9 (2.2%) of all births were with visible birth defect (Table 2).

**Table 2:** Gynecological, obstetric and nutritional characteristics of respondents in Central zone, Northern Ethiopia, 2018

Variables	Frequency	Percent
ANC visit		
Less than four	81	19.6
Four and above	332	80.4
Parity		
Primigravid	120	29.1
Multigravid	293	70.9
Gestational age at delivery (weeks)		
<37	53	12.8
37–42	355	86
42+	5	1.2
Counseling on additional diet		
Yes	287	69.5
No	126	30.5
Additional diet		
Yes	286	69.3
No	127	30.7
Pregnancy status		
Planned	383	92.7
Unplanned	30	7.3
Any medical disorder		
Yes	23	5.6
No	390	94.4
Hemoglobin level (md/dl)		
< 11	17	4.1
≥ 11	396	95.9
Height of mother		
≤ 1.49 m	41	9.9
1.50–1.55 m	80	19.4
≥ 1.55	292	70.7
MUAC (cm)		
< 21	96	23.2
21–23	75	18.2
≥ 23	242	58.6
Ambulance service		
Yes	251	60.8
No	162	39.2
Birth weight of live birth (g)		
< 2500	59	14.7
≥ 2500	341	85.3
Maturity of live birth		
Preterm	45	11.3
Term	355	88.7

#### Predictors of preterm birth

After the bivariate logistic regression, multivariate logistic regression was employed to identify the independent predictors of preterm birth among mothers delivered in the hospital. Accordingly, marital status (unmarried) (AOR = 5.21, 95% CI 1.8–15.075), mid upper arm circumference MUAC (< 11 cm) (AOR = 2.42, 95% CI 1.204–4.851) were found to be significantly associated with preterm birth among mothers delivered in the hospitals (Table 3).

**Table 3:** Factors associated with preterm birth among deliveries in Central zone, Northern Ethiopia, 2018

Variables	Pre-tem		COR	AOR
	Yes	No		
	n (%)	n (%)		
Age				
<20	9 (18.75)	39 (81.25)	1	1
20–35	35 (10.76)	290 (89.24)	0.52 (0.23–1.17)	0.76 (0.29–2.00
≥ 35	3 (7.50)	37 (92.50)	0.35 (0.09–1.40)	0.39 (0.06–2.82)
Income				
<2500	10 (9.90)	91 (90.10)	0.61 (0.27–1.39)	0.56 (0.212–1.50)
2500–5000	18 (9.62)	169 (90.38)	0.59 (0.30–1.18)	0.58 (0.26–1.27)
≥ 5000	19 (15.20)	106 (84.80)	1	1
Marital status				
Unmarried	6 (37.50)	10 (62.50)	5.21 (1.80–15.08)*	4.01 (1.17–13.71)**
Married	41 (10.32)	356 (89.68)	1	1
Maternal education				
Illiterate	5 (6.30)	74 (93.70)	0.46 (0.17–1.27)	0.45 (0.15–1.43)
Primary	21 (12.35)	149 (87.65)	0.96 (0.50–1.83)	0.85 (0.41–1.77)
Secondary and above	21 (12.80)	143 (87.20)	1	1
Maternal occupation				
House wife	20 (8.10)	227 (91.90)	1	1
Daily laborer	4 (21.10)	15 (78.90)	3.03 (0.92–10.00)	2.87 (0.76–10.79)
Employed	7 (11.70)	53 (88.30)	1.5 (0.60–3.73)	1.26 (0.43–3.68)
Merchant	12 (19.40)	50 (80.60)	2.72 (1.25–5.93)	2.3 (0.97–5.51)
Student	4 (16.00)	21 (84.00)	2.16 (0.68–6.92)	1.43 (0.39–5.21)
MUAC				
<21	20 (19.80)	81 (80.20)	2.62 (1.35–5.09)*	2.42 (1.20–4.85)**
21–23	6 (8.80)	62 (91.20)	1.03 (0.40–2.66)	1.1 (0.41–2.76)
≥ 23	21 (8.60)	223 (91.40)	1	1
Maternal height				
<150	6 (20.70)	23 (79.30)	2.2 (0.84–5.79)	2.15 (0.70–6.58)
150–155	7 (11.10)	56 (88.90)	1.06 (0.45–2.50)	1.36 (0.53–3.46)
≥ 155	34 (10.60)	287 (89.40)	1	1
ANC visit				
Less than four	15 (18.80)	65 (81.20)	2.17 (1.11–4.24)*	1.5 (0.69–3.29)
Four and above	32 (9.60)	301 (90.40)	1	1
Pregnancy status				
Planned	40 (10.50)	342 (89.50)	1	1
Unplanned	7 (22.60)	24 (77.40)	2.49 (1.01–6.15)*	1.18 (0.38–3.64)
Counseling additional diet				
Yes	25 (9.50)	237 (90.50)	1	1
No	22 (14.60)	129 (85.40)	0.96 (0.48–1.93)	0.57 (0.29–1.14)
Additional diet				
Yes	25 (9.70)	234 (90.30)	1	1
No	22 (14.30)	132 (85.70)	1.93 (1.02–3.64)*	1.09 (0.55–2.16)
Medical problem				
Yes	3 (7.50)	37 (92.50)	0.6 (0.18–2.05)	0.38 (0.05–3.10)
No	44 (11.80)	329 (88.20)	1	1

** Indicates statistically significant association at a p value < 0.05

### Discussion

Preterm birth remains a significant cause of morbidity and mortality among neonates and children. According to the 2016 Ethiopian Demographic health survey (EDHS), neonate mortality was 29 per 1000 live births. Among these causes of mortalities, preterm births were the major factor [17]. This facility-based study aimed to assess determinants of preterm births, in order to contribute to tackling morbidity and mortality related preterm births. According the findings of this study, prevalence of preterm birth were 12.8%. This preterm birth finding is similar to a study conducted at Brazil (12.3%), Kenya (12.3%) [18, 19]. But this finding is lower than the study of preterm at Oromia regional state Ethiopia, Bangladeshi, Nigeria, India, Zimbabwe, Malawi and several Northern European countries which shows 25.9%, 22.3%, 18.35, 15%, 16.4%, 16.8, 18.1% respectively [20–26] and little bit lower from the study done at Gondar university hospital and Tanzania which shows 14.2 and 14.3% respectively [15, 27]. This variation might be due to the difference in study area and period, improvements in the health care seeking behavior due to continuous education and improvements in the quality of health care provided to pregnant mothers. However, this finding is higher than the finding in Addis Ababa (7.1%), Debre Markos (11.6%) [28, 29]. It might be shown that our country Ethiopia brings change in health status of the mother and new born.

In the multivariable analysis marital status and MUAC measurement were significantly associated with preterm births. The study found that being unmarried increased the risk of preterm birth by four folds than the married. This finding is consistent with the findings done at Michigan [30]. This might be due to financial insecurity, psychosocial stress and low health care utilization.

This study also showed that MUAC less than 11 cm was significantly associated with preterm birth (PTB). The proportion of preterm birth was significantly higher (2.42 times) among mothers who had MUAC less than 11 cm compared to mothers with MUAC greater than or equal to 23 cm. This finding is consistent with the findings of studies done at Bangladeshi [31]. This might be maternal under nutritional status (mothers with MUAC < 11 cm) before and during pregnancy may contribute for preterm.

### Conclusion

The prevalence of preterm birth in this study was substantially high (12.8%). Factors like marital status that are unmarried and MUAC with less than 11 cm were associated with preterm birth. The factors identified in this study can be prevented and managed easily by providing appropriate care before pregnancy, antepartum and intrapartum period.

## Limitation of the study

The cross sectional nature of the data, which makes it impossible to draw inferences about the direction of relations among study variables and the data are retrospective and thus are subjected to recall bias.

## Data Availability

The datasets in which conclusion taken is available in the form of Microsoft Excel. It is available on requesting.

## Abbreviations

ANC: antenatal care
EC: Ethiopian Calendar
EDHS: Ethiopian Demographic and Health Survey
KM: kilometer
LBW: low birth weight
MUAC: Middle Upper Arm Circumference
MPH: Master of Public Health
MOH: Ministry of Health
RH: reproductive health
SPSS: Statistical Package for Social Science
USA: United States of America
WHO: World Health Organization
